# Safety assessment of nanomaterials using an advanced decision-making framework, the DF4nanoGrouping

**DOI:** 10.1007/s11051-017-3850-6

**Published:** 2017-05-09

**Authors:** Robert Landsiedel, Lan Ma-Hock, Karin Wiench, Wendel Wohlleben, Ursula G. Sauer

**Affiliations:** 1 Experimental Toxicology and Ecology, BASF SE, Carl-Bosch-Strasse 38, D-67056 Ludwigshafen, Germany; 2 Regulatory Toxicology, BASF SE, 67056 Ludwigshafen, Germany; 3 Advanced Materials Research, BASF SE, 67056 Ludwigshafen, Germany; 4 Scientific Consultancy—Animal Welfare, Hallstattfeld 16, 85579 Neubiberg, Germany

**Keywords:** Grouping, Hazard and risk assessment, Realistic exposure scenarios, Integrated approach for testing and assessment (IATA), In vitro effects, Nanomaterials, Societal implications

## Abstract

As presented at the 2016 TechConnect World Innovation Conference on 22–25 May 2016 in Washington DC, USA, the European Centre for Ecotoxicology and Toxicology of Chemicals (ECETOC) ‘Nano Task Force’ proposes a *Decision-making framework for the grouping and testing of nanomaterials* (DF4nanoGrouping) consisting of three tiers to assign nanomaterials to four main groups with possible further subgrouping to refine specific information needs. The DF4nanoGrouping covers all relevant aspects of a nanomaterial’s life cycle and biological pathways: intrinsic material properties and system-dependent properties (that depend upon the nanomaterial’s respective surroundings), biopersistence, uptake and biodistribution, and cellular and apical toxic effects. Use, release, and exposure route may be applied as ‘qualifiers’ to determine if, e.g., nanomaterials cannot be released from products, which may justify waiving of testing. The four main groups encompass (1) soluble, (2) biopersistent high aspect ratio, (3) passive, and (4) active nanomaterials. The DF4nanoGrouping foresees a stepwise evaluation of nanomaterial properties and effects with increasing biological complexity. In case studies covering carbonaceous nanomaterials, metal oxide, and metal sulfate nanomaterials, amorphous silica and organic pigments (all nanomaterials having primary particle sizes below 100 nm), the usefulness of the DF4nanoGrouping for nanomaterial hazard assessment was confirmed. The DF4nanoGrouping facilitates grouping and targeted testing of nanomaterials. It ensures that sufficient data for the risk assessment of a nanomaterial are available, and it fosters the use of non-animal methods. No studies are performed that do not provide crucial data. Thereby, the DF4nanoGrouping serves to save both animals and resources.

## Introduction

The traditional risk assessment paradigm is a hazard-driven approach that is based on a monocausal toxicological perspective (Jahnel 2015). While different ongoing initiatives aim at modernizing this traditional paradigm (Dix et al. 2007; Krewski et al. 2009; Tice et al. 2013; Rovida et al. 2015; Tluczkiewicz et al. 2016), it has been and is still being widely used for the risk assessment and regulation of substances (EP and Council of the EU 2006; Rudén and Hansson 2010). The traditional risk assessment paradigm is generally applicable to nanomaterials (Hankin et al. 2011; Anzai et al. 2012; ECHA 2012; Landsiedel 2015). Nevertheless, the full regulatory information requirements, e.g., in accordance with *Regulation (EC) No. 1907/2006 on the Registration, Evaluation, Authorisation, and Restriction of Chemicals* (REACH; EP and Council of the EU 2006), for every single variant of a given nanomaterial regarding particle size, shape, or surface properties (Stark et al. 2015) would lead to an insurmountable amount of testing. This would further stand in contradiction to the legal requirement to replace, reduce, and refine animal testing (3Rs principle; Russell and Burch 1959) that has been implemented in *EU Directive 2010/63/EU on the protection of animals used for scientific purposes* (EP and Council of the EU 2010).

Commencing risk assessments of nanomaterials offers the opportunity to apply modern concepts which are evolving for the general risk assessment of substances (Burden et al. 2017). In this respect, flexible and efficient approaches that allow identifying and collecting the data that are relevant for the safety assessment of nanomaterials are suggested. Aligning information needs to realistic exposure scenarios (indicated by a base set of information on exposure, fate/kinetics, and/or hazard) has been suggested as an important means to improve the risk assessment paradigm for nanomaterials (Bos et al. 2015; Oomen et al. 2014a, b, 2015; Hristozov et al. 2016; Sharma et al. 2016). In assessing nanomaterial exposure, it is distinguished between external and internal exposure. External exposure encompasses the release of particles over the life cycle of the respective products and aerosol concentrations in the air, for the inhalation route of exposure. Generally, external exposure is more complex for very small particles than that for larger sized particles. Humans are usually not exposed to a distinct nanoparticle but to a population of particles, aggregates, and agglomerates of different sizes, shapes, and surface coatings. Internal exposure refers to the dose of a nanomaterial that becomes systemically available via a given route of exposure.

The grouping of substances is widely recognized as an effective tool to streamline the collection of data for regulatory hazard and risk assessment. General grouping approaches for substances, regardless of their physical form, have been implemented in, e.g., the REACH Regulation. The European Chemicals Agency (ECHA 2013) describes grouping as the process of uniting substances into a common group *if they are structurally similar with physico-chemical, toxicological, ecotoxicological, and/or environmental fate properties that are likely to be similar or to follow a regular pattern*. Within a group, each individual substance may not need to be tested. Applying the grouping concept using read-across techniques, endpoint-specific effects of an unknown substance may be derived from the endpoint-specific effects of further substances within the group. The ECHA actively encourages the use of read-across under the REACH Regulation and has published a Read-Across Assessment Framework (RAAF) setting out the scientific principles for the scientific examination of read-across cases (ECHA 2017). Under REACH, any read-across approach must be based on structural similarity between the source and target substances. However, structural similarity alone is not sufficient to justify the possibility to predict property(ies) of the target substance by read-across. A read-across hypothesis needs to be provided. This hypothesis establishes why a prediction for a toxicological, ecotoxicological, or environmental fate property is possible and should be based on recognition of the structural aspects the chemical structures have in common and the differences between the structures of the source and target substances (ECHA 2017).

Specifically for the hazard and risk assessment (or grouping) of nanomaterials, a generally applied paradigm is still being developed (ECHA 2014; Arts et al. 2014). To provide a basis for regulatory provisions for nanomaterial hazard and risk assessment, different jurisdictions have laid down definitions of the term ‘nanomaterial’ (Boverhof et al. 2015). While the precise components of these definitions vary, all of them are based on material characteristics. However, the underlying nanomaterial properties are neither monocausal nor linearly related to the hazard of nanomaterials (Lynch et al. 2014). Basing nanomaterial hazard assessment on such material properties alone is likely to result in the over- or underestimation of hazards or failure to recognize relevant hazards at all (Arts et al. 2014).

To account for nanomaterial complexity, the grouping and hazard assessment of nanomaterials should address all relevant aspects of a nanomaterial’s life cycle and biological pathway from its release up until (potential) apical effects (Braakhuis et al. 2016). Similar to adverse outcome pathways (AOPs; Ankley et al. 2010), biological pathways may encompass a multitude of interlinked steps that are not necessarily already fully understood for each and every type of nanomaterial. Nevertheless, nanomaterial grouping does not require that all pieces of knowledge concerning the respective steps are available. Relevant and quantifiable aspects for nanomaterial grouping include intrinsic material properties and system-dependent properties, specific types of use and exposure, uptake and kinetics, and early cellular and apical effects (Landsiedel et al. 2010; Arts et al. 2014; Oomen et al. 2014a, 2015; Stone et al. 2014; Braakhuis et al. 2016).

- *Intrinsic material properties* are defined as properties that do not change easily by the environment that surrounds the materials during the measurement, e.g., chemical composition or primary particle size and shape. If intrinsic properties change after contact of the material to specific environments, this is considered a ‘transformation’ of the nanomaterial, and it is often irreversible. As described by Graham et al. (2017), continuous physico-chemical transformations of the nanoparticles, the so-called bio-processing, are observed in biological systems.
- *System-dependent properties* are defined as properties that change easily, often reversibly, by the (nano)material during measurement (Luoto et al. 1994; Potthoff et al. 2015; Kettler et al. 2016). The environment may be a specific product matrix, a cell culture medium, the lung-lining fluid, or blood. Accordingly, system-dependent properties include dissolution, dispersibility, and surface reactivity.

Risk is the product of hazard and exposure. Clearly, exposure varies during the life cycle of a nanomaterial (e.g., composites with nanomaterials embedded in a matrix). While the apical effects of a nanomaterial are eventually directed by its intrinsic material properties, they are the result of several preceding steps, including uptake, distribution, nano-bio interactions (often termed system-dependent properties or functionality), and cellular effects. The exact correlation between a nanomaterial’s intrinsic material properties and its apical effect may not be obvious. In these cases, nanomaterial grouping should take into account ‘functionalities’ rather than relying on intrinsic material properties alone. Functionalities include system-dependent material properties, in vitro effects, and release and exposure (Arts et al. 2014, 2015, 2016; Braakhuis et al. 2016; Oomen et al. 2015).

Different regulatory authorities and international research consortia have published approaches for the specific grouping of nanomaterials, and preliminary guidance is provided in the context of substance-related legislation or in the occupational setting. An extensive review conducted in 2014 by the European Centre for Ecotoxicology and Toxicology of Chemicals (ECETOC) ‘Nano Task Force’ revealed that the available approaches for the grouping of nanomaterials already go beyond the determination of mere structure-activity relationships and are founded on different aspects of the nanomaterial’s life cycle or biological pathway (Arts et al. 2014). For instance, material properties and biophysical interactions are addressed in the categorization scheme of the German Federal Institute for Occupational Safety and Health (BAuA 2013, 2015; Packroff and Gebel 2014). Nanomaterial exposure is a fundamental component of the grouping concept of the US National Institute for Occupational Safety and Health (Kuempel et al. 2012; DHHS (NIOSH) 2012). Defined mechanisms of toxic effects of nanomaterials form the basis of the grouping and testing approaches published by Lai (2012), Wang et al. (2014), or Nel et al. (2013, 2015). However, none of the available approaches cover all relevant aspects of a nanomaterial’s life cycle and biological pathway, and most of them have not advanced beyond theoretical and conceptual stages (Arts et al. 2014).

## Decision-making framework for the grouping and testing of nanomaterials

As presented at the 2016 TechConnect World Innovation Conference on 22–25 May 2016 in Washington DC, USA, the ECETOC Nano Task Force developed a comprehensive functionality-driven concept for the grouping of nanomaterials (Arts et al. 2015, 2016). This *Decision-making framework for the grouping and testing of nanomaterials* (DF4nanoGrouping) consists of three tiers to assign nanomaterials to four main groups (MGs), to perform subgrouping within the MGs, and to determine and refine specific information needs. Addressing all relevant aspects of a nanomaterial’s life cycle and biological pathway, the essential grouping criteria include intrinsic material properties in Tier 1 (water solubility, particle morphology, and chemical composition) and system-dependent properties in Tier 2 (dissolution in biological media, surface reactivity, particle dispersibility, and in vitro effects). In Tier 3, the Tier 1 and Tier 2 MG assignment that is based upon non-animal testing alone is confirmed or corrected using data from in vivo short-term studies. For the inhalation route of exposure, i.e., the predominant route of uptake for most nanomaterials (Landsiedel et al. 2012a), the rat short-term inhalation study (STIS; Ma-Hock et al. 2009a; Landsiedel et al. 2014a) is recommended.

The four MGs of the DF4nanoGrouping encompass (MG1) soluble nanomaterials, (MG2) biopersistent high aspect ratio (HAR) nanomaterials, (MG3) passive nanomaterials, and (MG4) active nanomaterials (cf. Table 1 for grouping criteria and threshold values). Prior to the application of the DF4nanoGrouping tiers, further intrinsic material properties, such as the ‘as manufactured’ surface area and surface charge (specified as ‘supplementary criteria’ in Table 1), may be used to define if a substance is in fact a nanomaterial, e.g., in accordance with the EU recommendation (EU Commission 2011). Nevertheless, the use of such additional intrinsic properties to identify a nanomaterial and different nanoforms of a substance does not compromise their subsequent grouping by functionality-driven properties for the purpose of hazard assessment.

**Table 1:** DF4nanoGrouping: grouping criteria, threshold values, and relation to main group assignment (adapted from Arts et al. 2015, 2016)

DF4nanoGrouping Tier	Grouping criterion	Threshold value for grouping	Main group (MG) assignment or indication	Benchmark material; if applicable
Tier 1 Intrinsic material properties	Water solubility	>100 mg/L [a]	Assignment to MG1	ZnO NM-110 and NM-111, 10 nm-CuO: limited water solubility TiO_2_ NM-105: low water solubility
	Particle size and shape	Aspect ratio > 3:1, length > 5 μm, diameter < 3 μm [b]	Indication for MG2	
	Composition including impurities	≥0.1% of component with GHS classification for systemic effects	Indication for MG4	
Tier 2 System-dependent properties In vitro effects	Dissolution in biological fluids	>100 mg/L [a]	Globular nanomaterials: >100 mg/L: Indication for MG1 Fibers: <100 mg/L: indication for MG2	10 nm-CuO: high dissolution
	Surface reactivity	≥10% of Mn_2_O_3_ reactivity, which is equal to: ≥0.19 μU FRAS/m^2^ × h	Assignment to MG4	Quartz dust DQ12: high surface reactivity BaSO_4_ NM-220: not oxidative
	Dispersibility	AAN <3 or diameter < 100 nm	Assignment to MG2 or MG4, as applicable	a-SiO_2_-susp with acrylate surface functionalization: dispersible a-SiO_2_-susp without surface functionalization: agglomeration
	Cellular effects	Effect at ≤10 μg/cm^2^ [c]	Assignment to MG4	ZnO NM-110 and NM-111: activity (shedding of ions) CeO_2_ NM-211 and NM-212: activity BaSO_4_ NM-220: passivity
Tier 3 In vivo screening	Toxic potency	STIS NOAEC; four ranges: I: <0.5 mg/m^3^ and no regression or progression of effects II: <1 mg/m^3^; III: <10 mg/m^3^ IV: ≥10 mg/m^3^	Ranges I–III: Confirmation of MG2 or MG4; subgrouping of MG4; Range IV: Confirmation of MG3	MWCNT NM-400: Range I CeO_2_ NM-211 and NM-212: Range II TiO_2_ NM-105; a-SiO_2_-susp without surface functionalization: Range III BaSO_4_ NM-220: Range IV
	Biopersistence	t_50_ < 40 days	Confirmation of MG1	CeO_2_ NM-211 and NM-212: decelerated clearance TiO_2_ NM-105: physiological clearance BaSO_4_ NM-220: accelerated clearance
Qualifier	Dustiness	None assigned	Indication of a substance’s emission potential	
Supplementary criteria	Surface area	None assigned	Not primary grouping criteria	
	Surface chemistry	None assigned	Not primary grouping criteria	
	Surface charge	Positive: ζ >10 mV	Joint evaluation with ‘dispersibility’ [d] Pos. surface charge: indication for MG4	CeO_2_ NM-211 and NM-212: positive surface charge
	Hydrophobicity	None assigned	Joint evaluation with ‘dispersibility’; cf. also Hofmann et al. (2017) [d]	

*Abbreviations*: *AAN* average agglomeration number, *a-SiO*_*2*_ *-susp* suspended amorphous SiO_2_ (Levasil® 200), *FRAS* ferric reducing ability of serum, *GHS* globally harmonized system, *MG* main group, *NM* nanomaterial, *NOAEC* no observed adverse effect concentration, *STIS* short-term inhalation study

Based upon the outcome of the case studies, Arts et al. (2016) laid down and justified the following adaptations to the threshold values that had been established in Arts et al. (2015):

[a] While the threshold values for water solubility and dissolution are adequate for nanomaterials that release ions with GHS classification for systemic effects, they may have to be reconsidered for substances that dissolve into non-toxic components

[b] Nanomaterials may be assigned to MG2 on account of high aspect ratio, fiber diameter, and insolubility/low dissolution in water or biological media, even though their length does not meet the World Health Organization criterion (>5 μm)

[c] This threshold value applies for cytotoxicity tests performed with lung epithelial cells. For in vitro assays performed with alveolar macrophages, a particle surface area-based threshold value of 6000 mm^2^/mL is laid down (Wiemann et al. 2016)

[d] ‘Joint evaluation’ implies that strong nanoparticle surface charge, and especially strong negative surface charge, leads to low opsonization, which then leads to potentially higher mobility, e.g., quantum dots (Choi et al. 2010), SiO_2_.acrylate (Arts et al. 2016), and ZrO_2_.acrylate (Landsiedel et al. 2014a). Since dispersibility is assessed directly as an essential grouping criterion, surface charge does not need to be assessed separately

Use (including manufacture), release, and route of exposure are applied as ‘qualifiers’ within the DF4nanoGrouping to determine relevant exposure scenarios (Bos et al. 2015; Sharma et al. 2016). If nanomaterials cannot be released from a product matrix in any way (Wohlleben et al. 2011a; Bräu et al. 2012; Ding et al. 2017), these qualifiers may serve to justify the waiving of testing (Arts et al. 2015).

For each grouping criterion and the qualifier release, Arts et al. (2015, 2016) suggest pragmatic methods, many of which are standardized (Wohlleben et al. 2013; Potthoff et al. 2015). Additionally, specific threshold values for nanomaterial assignment to one of the MGs and benchmark materials, predominantly from the Organisation for Economic Co-operation and Development (OECD) sponsorship program (OECD 2010), are suggested (Table 1). (Throughout this article, all NM-x numberings (e.g., ZnO NM-110) relate to the codes of representative nanomaterials of the OECD sponsorship program.)

Nevertheless, the DF4nanoGrouping does not imply fixed testing schemes for all nanomaterials in all applications. On the contrary, such ‘tick-box’ testing may result in the collection of scientifically unnecessary information. Instead, functionality-driven grouping, as proposed in the DF4nanoGrouping, is closely linked to (or even undistinguishable from) integrated approaches for the testing and assessment (IATAs) of nanomaterials. Both of these processes take into account the life cycle of a nanomaterial and its biological pathway, and both support a concern-driven stepwise collection and evaluation of information that are relevant for the given purpose (Oomen et al. 2014a; Stone et al. 2014). The collection of data is concluded as soon as the hazard and exposure potential and risk of a given nanomaterial can be assessed.

The tiered framework and four MGs of the DF4nanoGrouping stand in line with the requirements and stipulations from different organizations, authorities, and research groups, such as the International Standardization Organization (ISO 2014); the United States Environmental Protection Agency (EPA 2015), the German Environmental Protection Agency (UBA 2014), researchers from the German Federal Institute for Occupational Safety and Health (Gebel et al. 2014), or the Dutch National Institute for Public Health and the Environment (RIVM; Sellers et al. 2015).

Braakhuis et al. (2016) caution that grouping frameworks, such as the DF4nanoGrouping, will most likely and for most cases not be sufficient to perform a risk assessment and fully demonstrate safe use. In addressing such concerns, the ECETOC Nano Task Force conducted case studies to evaluate the usefulness of the DF4nanoGrouping for hazard and risk assessment (focusing on the inhalation route of exposure) and to further refine it as necessary (Arts et al. 2016). In these case studies, a broad spectrum of economically relevant inorganic nanomaterials was assessed, covering carbonaceous nanomaterials, metal oxide and metal sulfate nanomaterials, amorphous silica nanomaterials, non-nanosized and nanosized organic pigments, and non-nanosized crystalline quartz dust. Since a multitude of different uses is foreseeable for most of these nanomaterials, the case studies did not take into account intended uses or specific exposure and release scenarios.

An overview of the outcome of the DF4nanoGrouping case studies is provided in Table 2. Altogether 22 of the 25 test materials fitted into the four MGs based on the non-animal Tiers 1 and 2 alone. For the other three materials, the hazard was overpredicted in the non-animal tiers, i.e., they indicated a concern that was not confirmed in Tier 3 in the in vivo STIS. Hazard was never underpredicted in the non-animal tiers. While 90-day studies were not available for all test materials, the outcome of the available subchronic studies stood in accordance with the MG assignments (Arts et al. 2016).

**Table 2:**
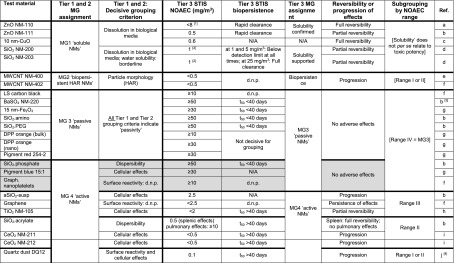
Nanomaterial assignment to main groups applying the DF4nanoGrouping non-animal Tiers 1 and 2 and Tier 3 STIS biopersistence or toxic potency (NOAEC), respectively (adapted from Arts et al. 2016)

*Abbreviations*: *d.n.p*. determination not possible for technical reasons, *HAR* high aspect ratio, *LS* low surface, *MG* main group, *N/A* not available, *NOAEC* no-observed adverse effect concentration, *STIS* rat short-term inhalation study

STIS data were retrieved from the following sources: [a] Bellmann (2011); [b] Landsiedel et al. (2014a); [c] Gosens et al. (2015); [d] Arts et al. (2007); [e] Ma-Hock et al. (2009b); [f] Ma-Hock et al. (2013) and Treumann et al. (2013); [g] Hofmann et al. (2016); [h] Ma-Hock et al. (2009a); [i] Keller et al. (2014); [j] Henderson et al. (1995)

The STIS NOAEC ranges correspond to: Range I: <0.1 mg/m^3^; Range II: <1 mg/m^3^; Range III: <10 mg/m^3^; Range IV: ≥10 mg/m^3^

Color legend: Gray shading: In Tier 2, SiO_2_.phosphate was assigned to MG4 on account of its high dispersibility, Pigment blue 15:1 was assigned to MG4 on account of its activity in the in vitro alveolar macrophage assay, and graphite nanoplatelets were assigned to MG4 since determination of surface reactivity was not possible for technical reasons. In Tier 3, high STIS NOAEC (Range IV) are recorded for all three substances indicating MG3 ‘passivity’

[1] 14-day exposure, only one test substance concentration (i.e., 8 mg/m^3^)

[2] For equivalent substance

[3] Furthermore, there are strong indications that BaSO_4_ is at least partially soluble in vivo after inhalation (Konduru et al. 2014)

Consequently, the DF4nanoGrouping proved efficient in sorting out nanomaterials that could undergo hazard assessment without additional testing. These are soluble nanomaterials (MG1) whose further hazard assessment should rely on read-across to the dissolved materials; HAR nanomaterials (MG2) which may be assessed according to their potential fiber toxicity (Cassee et al. 2015); and passive nanomaterials (MG3) that do not elicit material-specific effects, but only cause effects in rats under pulmonary overload conditions: In the rat, inhaled particles have been observed to damage lung cells, especially if the macrophages’ clearance capacity is overwhelmed. The clearance capacity is impaired once 6% of the macrophage volume in the rat lung has been filled, and macrophage stasis occurs at 60% filling of the macrophage volume (Morrow 1988, 1994; Morrow et al. 1996). Therefore, determination of the lung burden and clearance rates of inhaled nanoparticles is especially relevant to determine whether pulmonary particle overload in the rat lung is likely, which may further lead to particle uptake and hence biodistribution (Kuempel et al. 2014). However, the human health relevance of lung tumors observed in the rat upon pulmonary particle overload conditions is at least questionable (ECETOC 2013).

By sorting out nanomaterials that could undergo hazard assessment without additional testing, the DF4nanoGrouping allows identifying MG4 active nanomaterials. These are the nanomaterials that merit in-depth investigations. The DF4nanoGrouping provides a solid scientific rationale to subgroup MG4 active nanomaterials which allows determining the specific information needs for the in-depth investigations (Arts et al. 2016).

The following Sections provide:

- details on the determination of cellular effects, an essential grouping criterion of Tier 2 of the DF4nanoGrouping;
- a discussion of nanomaterial hazard assessment by apical effects upon inhalation exposure and means to subgroup MG4 active nanomaterials by information obtained in the Tier 3 STIS;
- a summary of aspects of internal exposure to nanomaterials as relevant information to streamline testing needs during nanomaterial grouping;
- a brief outlook on nanomaterial hazard assessment upon long-term exposure and upon oral, dermal or local exposure;
- an outline on how the DF4nanoGrouping may be applied for read-across approaches.

## Determination of cellular effects for nanomaterial hazard assessment

Nanomaterials may induce cellular effects by a number of different mechanisms of toxicity, i.e., (1) membrane damage including cationic phagolysosome damage that may ultimately lead to apoptosis and autophagy; (2) generation of reactive oxygen species (ROS), oxidative stress, redox activities, and photo-catalytic effects; (3) inflammasome activation and cytokine and chemokine production; (4) the cytotoxic effects of toxic ions; (5) fiber effects; and (6) DNA damage (Nel et al. 2013, 2015; Landsiedel et al. 2009, 2014b; Park et al. 2015; Stern et al. 2012). There is no indication that nanomaterials elicit hitherto unknown cellular effects.

Numerous in vitro studies have been published evaluating the effects of nanomaterials on cells or tissues derived from the respiratory tract. As reviewed by Landsiedel et al. (2014b), these in vitro studies applied a multitude of test systems, cell culture conditions, exposure durations, and endpoint detection methods and methods for test substance preparation. Nanomaterial concentrations ranged from a few micrograms per milliliter to several milligram per milliliter. To date, standardized in vitro assays to assess the cellular effects of nanomaterials, let alone guidance on how to incorporate in vitro assays into the hazard and risk assessment of nanomaterials, are unavailable (Kroll et al. 2011; Landsiedel et al. 2014b). Only few in vitro studies investigating nanomaterial pulmonary toxicity aim at predicting hazard potency (Cho et al. 2013; Landsiedel et al. 2014b; Wiemann et al. 2016).

The outcome of in vitro assays may be adulterated when nanomaterials interfere with assay reagents. Nanomaterials may bind to the marker enzyme lactate dehydrogenase (LDH; Wohlleben et al. 2011b) or they may interact with dyes and dye products, including neutral red and the tetrazolium salt MTT (Monteiro-Riviere et al. 2009). Such interferences may be recognized and corrected for, e.g., by including cell-free controls in the in vitro studies. Moreover, in many published in vitro studies, the effective dose (the particle mass reaching the cultured cells) was neither calculated nor measured, and in vitro doses were rarely correlated to aerosol concentrations or lung burdens in inhalation studies (Cohen et al. 2015; Kettler et al. 2016). Even though many nanomaterials induced some form of inflammatory and/or cytotoxic reaction in vitro, these effects were often only observed at concentrations that were much higher than those which could be achieved by in vivo experiments and do not reflect realistic exposure scenarios (Kroll et al. 2011; Landsiedel et al. 2014b).

If in vitro assays use standardized protocols for test substance preparation and assay performance and are conducted at concentrations reflecting effective in vivo dosages (Cohen et al. 2015), they allow assessing mechanisms of nanomaterial toxicity (Horev-Azaria et al. 2013). Improved methods to estimate the effective dose of nanomaterials in vitro have been proposed (Hinderliter et al. 2010; DeLoid et al. 2014).

Recently, the in vitro alveolar macrophage assay has proven highly predictive of in vivo respiratory tract effects (Wiemann et al. 2016). This assay uses rat NR8383 alveolar macrophages which are similar to alveolar macrophages in the rat lung that sequester the vast majority of inhaled particles upon short-term exposure. The in vitro NR8383 alveolar macrophage assay jointly assesses the four parameters cellular release of (1) LDH, (2) glucuronidase, (3) tumor necrosis factor alpha, and (4) ROS. Wiemann et al. (2016) calculated the particle surface area-based range of <6000 mm^2^/mL as reflecting in vitro cellular ‘non-overload’ conditions and showed that it corresponds to the STIS non-overload range. Significant effects observed below this threshold are interpreted as material-specific biological effects that are not merely induced by cellular overload conditions. Test materials are assessed as active if at least two of the four abovementioned parameters undercut the threshold, and they are assessed as passive if none or one parameter is altered (Wiemann et al. 2016).

A total of 18 inorganic nanomaterials and two nanosized organic pigments was evaluated in the NR8383 alveolar macrophage assay, and the outcomes were compared to the DF4nanoGrouping Tier 3 MG3/MG4 categorization, by which a STIS no-observed adverse effect concentration (NOAEC) ≥10 mg/m^3^ indicates MG3 passive nanomaterials, whereas a NOAEC <10 mg/m^3^ indicates MG4 active nanomaterials. This threshold was set empirically based upon the NOAEC recorded in more than 30 STIS (Klein et al. 2012; Ma-Hock et al. 2013; Landsiedel et al. 2014a)**.**

For all but one test material, where the in vitro data suggested a concern that was not confirmed in vivo*,* the NR8383 alveolar macrophage assay correctly predicted the STIS-based distinction between passive and active nanomaterials (Wiemann et al. 2016). In conclusion, subject to its further validation, the in vitro NR8383 alveolar macrophage assay appears suitable to distinguish MG4 active from MG3 passive nanomaterials. Thereby, it may serve to determine whether in vivo inhalation testing is necessary for hazard and risk assessment or not (Arts et al. 2016; Wiemann et al. 2016).

For none of the nanomaterials evaluated in the DF4nanoGrouping case studies, evidence of in vivo *genotoxicity* is available, and possibly occurring in vitro genotoxic effects do not correlate to the outcomes of in vivo studies. Hence, threshold values or benchmark materials for in vitro or in vivo genotoxicity were not established in DF4nanoGrouping (Arts et al. 2015, 2016). Nevertheless, assessment of a material’s genotoxic potential forms an important pillar of hazard assessment. As also stands true for the hazard assessment of non-nanosized chemicals, the employment of nanomaterial genotoxic effects for hazard assessment may be enhanced when the underlying mechanisms are known. However, the elucidation of the potentially complex mechanisms of genotoxic effects may be challenging. A practical and pragmatic solution may be the application of a battery of standard testing methods covering a wide range of mechanisms. For this purpose, the available standard genotoxicity test methods may require adaptations to ensure applicability for nanomaterials, and the interpretation of test results may require additional considerations, taking into account, e.g., effective in vitro dosages or specific system-dependent material properties of the nanomaterials (Landsiedel et al. 2009, 2014b; Oesch and Landsiedel 2012; Pfuhler et al. 2013; Valsami-Jones and Lynch 2015; Maser et al. 2015).

## Nanomaterial hazard assessment by apical effects upon inhalation exposure

For the inhalation route of exposure, the DF4nanoGrouping recommends the rat STIS in Tier 3 for those nanomaterials that are assessed as MG4 active in the non-animal Tiers 1 and 2. (Also, rat intratracheal instillation studies may be useful to rank the harmful effects of nanomaterials (Morimoto et al. 2015).) The STIS is essentially an adaptation of the OECD test guideline (TG) 412 “Subacute inhalation toxicity: 28-day study.” The STIS protocol foresees 5 days of exposure (6 h/day) and a mandatory post-exposure observation period of 4 to 13 weeks; it includes appropriate aerosol generation and characterization, bronchoalveolar lavage parameters, and lung burden assessments (Arts et al. 2007; Ma-Hock et al. 2007, 2009a; Landsiedel et al. 2014a). Thereby, the STIS allows reducing and refining the use of animals as compared to the traditional OECD TG 412 (Burden et al. 2017). It allows determining the nanomaterials’ potential to elicit effects in the respiratory tract, i.e., the primary site of contact, and it provides information on the test materials’ toxic potency, and the location and reversibility of effects. Moreover, assessment of lung burden and material translocation to extra-pulmonary tissues provide preliminary biokinetic information (Landsiedel et al. 2014a).

Table 2 summarizes rat STIS data for the DF4nanoGrouping case study substances that were previously published by Arts et al. (2007), Ma-Hock et al. (2009a, b, 2013), Bellmann (2011), Treumann et al. (2013), Landsiedel et al. (2014a), Keller et al. (2014), Gosens et al. (2015), and Hofmann et al. (2016). Additionally, Ma-Hock et al. (2012) reported that up to 10 mg/m^3^ acrylic ester polymers containing different fractions of nanoparticles did not elicit any adverse effects in the rat STIS.

Nanomaterials assigned as MG4 active in Tier 3 of the DF4nanoGrouping encompass graphene, TiO_2_, CeO_2_, and suspended amorphous SiO_2_ (without surface functionalization and with acrylate-based surface functionalization). The NOAEC values of these active nanomaterials cover a broad range of aerosol concentrations, i.e., all three STIS NOAEC ranges allocated to MG4 (Range I <0.1 mg/m^3^; Range II <1 mg/m^3^; Range III <10 mg/m^3^, cf. Tables 1 and 2). This broad range of NOAEC values underlines that the hazard and risk assessment of active nanomaterials may require further in-depth investigations. The DF4nanoGrouping foresees subgrouping active nanomaterials by the respective STIS NOAEC range. Furthermore, active nanomaterials (MG4) can be subgrouped by the reversibility or progression of effects and by their pattern of biodistribution. Such subgrouping may serve to identify and refine specific testing needs.

In subgrouping MG4 active nanomaterials by pattern of biodistribution, Arts et al. (2015, 2016) distinguish between nanomaterials that only become available in the primary organ (i.e., the respiratory tract for the inhalation route of exposure), nanomaterials that are additionally found in the mononuclear phagocyte system (MPS), and, finally, nanomaterials that become systemically available outside the MPS (above one mass percentage of the total dose, each). Given the limited number of potential modes-of-action of nanomaterials in the lung, most of the testing needs beyond Tier 3 of the DF4nanoGrouping are expected to arise from indications of extra-pulmonary effects or the need to consider for distinct biokinetics due to increased lung deposition or prolonged clearance.

## Estimation of nanomaterial internal exposure

For none of the case study materials, systemic availability outside the MPS was recorded in the STIS (Arts et al. 2016). Nevertheless, clearly, the biokinetics of nanomaterials, i.e., biopersistence, systemic uptake, and biodistribution, should be addressed during risk assessment since they may affect the internal exposure to a nanomaterial, i.e., the dose present in a given organ over time (Braakhuis et al. 2016).

The *biopersistence* of a nanomaterial, i.e., its property to persist in a cell, tissue, organ or organism, may affect its retention and clearance (and hence organ burden) as well as systemic uptake from the primary site of contact and biodistribution. In vivo, nanomaterials may be non-soluble or poorly soluble (i.e., biopersistent) or of moderate or high solubility. The toxicity of readily soluble nanomaterials is expected to be dominated by the dissolved ions, and particle- or fiber-like toxicity is largely expected for biopersistent particles. The relevance of nanomaterial biopersistence for hazard and risk assessment is underlined by the fact that this parameter is essential for nanomaterial assignment to MG1 and MG2 of the DF4nanoGrouping. While MG1 soluble nanomaterials have a low in vivo biopersistence, the MG2 for biopersistent HAR nanomaterials is not only based upon a HAR but also on the fibers’ high biopersistence.

Determination of the *lung burden* of inhaled nanoparticles over time is especially relevant to determine whether pulmonary particle overload in the rat is likely, which may lead to lung cell damage and systemic uptake (Morrow 1988; Kuempel et al. 2014; Cassee et al. 2015). As discussed above the human health relevance of lung tumors observed in the rat under pulmonary particle overload conditions is at least questionable (ECETOC 2013).

Interestingly, for all MG3 passive nanomaterials for which rat lung burden data were available for the DF4nanoGrouping case studies, pulmonary half-lives below 40 days were recorded, whereas for all MG4 active nanomaterials for which lung burden data were available, the pulmonary half-lives exceeded 40 days (Table 2). While this observation points to the impact of biopersistence on the evolvement of pulmonary effects, knowledge on specific nanomaterial properties that eventually affect biopersistence is just beginning to evolve. Graham et al. (2017) highlight that dose-response relationships are complicated by the continuous physico-chemical transformations of nanoparticles. For technical reasons, no lung burden data were available for the carbonaceous case study substances, i.e., MG2 multi-walled carbon nanotubes, MG3 low surface carbon black and graphite nanoplatelets, and MG4 graphene. Specific (e.g., radioactivity-based) methodologies are required to investigate the intrapulmonary biokinetics of such carbonaceous substances.

Apart from the degree of biopersistence or particle dissolution (Konduru et al. 2014; Yokel et al. 2014), cumulative lung burden appears dependent upon the size of agglomerated particles and the likelihood of their disintegration (Konduru et al. 2014; Gebel et al. 2014).

Agglomerates that form in aerosols and do not disintegrate in the lung are unlikely to become systemically available (Landsiedel et al. 2014a; Konduru et al. 2014). Overall, lung burden in rats upon short-term inhalation exposure is low (Morfeld et al. 2012) and depends upon aerosol concentration, exposure duration, deposition efficiency, and lung clearance. Typically, short-term exposure (5 days) with aerosol concentrations up to 50 mg/m^3^ resulted in lung burdens ranging from approximately 1% of the total amount of inhaled particles for surface-functionalized ZrO_2_ to approximately 10% of the inhaled dose for CeO_2_ nanomaterials immediately after 5 days of exposure (Landsiedel et al. 2014a). (For 50 mg/m^3^ (target concentration) of ZrO_2_ with acrylate surface functionalization, the entire amount of particles was 18 mg,^1^ 0.17 mg of which was the lung burden immediately after the exposure period; 0.9% of the total inhaled particle mass was deposited in the lung. For 10 mg/m^3^ (target concentration) of CeO_2_ nanomaterial, the entire inhaled amount was 4.2 mg, of which 0.4 mg was measured as lung burden immediately after the exposure period.)

Particle deposition and retention in the lung may affect apical effects. Upon inhalation exposure to CeO_2_ NM-211, the dose rate of CeO_2_ deposition drove an initial neutrophil-dominated inflammatory reaction (Keller et al. 2014). During 4 weeks of exposure, cell counts shifted to a macrophage-dominated inflammation that progressed towards a granulomatous reaction depending on the duration and amount of particles retained in the lung (Keller et al. 2014; Pauluhn 2014).

Typically, *systemic uptake* of nanomaterials upon inhalation is very low and lies in ranges below 1% of the dose retained in the lung. Further, systemic uptake may occur especially under high-dose conditions (Gebel et al. 2014; Moreno-Horn and Gebel 2014). Generally, a relevant different translocation rate of nanomaterials as compared to their bulk counterparts has not been observed (Moreno-Horn and Gebel 2014). If taken up systemically, nanoparticles are prone to lymphatic transport, but they may also be translocated with the circulatory system (Albanese et al. 2012; Landsiedel et al. 2012b). By contrast, absorption via the olfactory system, if it occurs, does not seem to be specific for the nanosized variants of a material (Moreno-Horn and Gebel 2014; Oberdoerster et al. 2009).

Also, nanoparticles that enter the blood stream are mostly taken up by the MPS that acts as a depot for nanoparticles (Fabian et al. 2008). This explains observed extra-pulmonary accumulations in the liver and spleen, which are the first organs that particles circulating in the blood encounter. Just as lung burden, secondary organ burden is also dependent upon nanomaterial transport to and clearance from the respective organs.

## Nanomaterial hazard assessment upon long-term exposure

Repeated exposure to nanoparticles may lead to tissue accumulation if exposure levels and systemic availability are high enough, as is also known for bulk substances (Gebel et al. 2014; Moreno and Gebel 2014; Oomen et al. 2014a; Yokel et al. 2014). Nevertheless, in an extensive review, Moreno-Horn and Gebel (2014) found no convincing evidence for systemic toxicity of ‘granular biodurable particles without specific toxicity’ (GBPs; or ‘poorly soluble low toxicity particles’ (Kuempel et al. 2012, 2014)).

An extensive meta-analysis of chronic rat inhalation studies with GBPs found the difference in *carcinogenic potency* between nanosized and micron-sized GBPs to be low (i.e., a factor of 2.0–2.5 referring to mass concentration) (Gebel 2012). However, data are sparse whether nanomaterials, upon long-term low-dose administration, may accumulate to an extent that chronic adverse effects may evolve, just as there are limited data on the chronic effects of inhaled nanomaterials (Becker et al. 2011; Gebel et al. 2014; Moreno-Horn and Gebel 2014). A 2-year combined chronic toxicity-carcinogenicity study performed according to OECD TG 453 assessing BaSO_4_ NM-220 (MG3) and CeO_2_ NM-212 (MG4) is expected to be completed in 2017 (Landsiedel et al. 2016; Ma-Hock et al. 2016; Groeters et al. 2017).

Previously, lung carcinogenicity has been shown in the rat upon chronic exposure to high concentrations of two nanosized GBPs (that are assigned to MG4), i.e., 10 mg/m^3^ TiO_2_ and 10 mg/m^3^ high surface carbon black (Heinrich et al. 1995) as well as 6.5 mg/m^3^ high surface carbon black (Nikula et al. 1995). Two-year exposure to extremely high 250 mg/m^3^ non-nanosized TiO_2_ in rats elicited the formation of bronchioloalveolar adenomas and cystic keratinizing squamous cell carcinomas, whereas concentrations of up to 50 mg/m^3^ of this test substance did not (Lee et al. 1985). Lee and co-workers questioned the human health relevance of these observations since the recorded lung tumors were different from common human lung cancers in terms of tumor type, anatomic location, and tumorigenesis and were devoid of tumor metastasis (Lee et al. 1985). Two-year exposure to 2 mg/m^3^ of the multi-walled carbon nanotube MWCNT-7 (selected as benchmark material for MG2 biopersistent HAR nanomaterials in the DF4nanoGrouping) elicited lung carcinomas without pleural mesothelioma in rats, with lung carcinomas in male rats additionally being observed at aerosol concentrations of 0.2 mg/m^3^ (Kasai et al. 2016).

By comparison, none of the epidemiological studies examining workers exposed to nanosized carbon black or TiO_2_ provide evidence of carcinogenicity in humans (IARC 2010a; MAK 2013). Lung tumor formation in rats upon long-term exposure to high levels of GBPs is thought to result from reduced particle clearance involving chronic inflammation associated with oxidative stress, secondary genotoxicity, and cell proliferation (Greim and Ziegler-Skylakakis 2007). It remains unclear why rats develop lung tumors, whereas some other animal species (including humans) do not. Based upon an AOP approach published by ECETOC (2013), Morfeld et al. (2015) elaborate on the cascade of GBP-initiated cellular and molecular events that lead to pretumor conditions in the lung. In step 1, inflammation-promoting mediators are produced; step 2 involves an increased production of anti-inflammatory mediators; step 3, injury repair; and step 4, intermediate endpoints including DNA mutations and cell proliferation. Following inhalation exposure to high concentrations of GBPs, parameters reflecting these four steps of the AOP were much more strongly affected in rats than those in mice, hamsters, or humans (Bermudez 2004; IARC 2010b; ECETOC 2013; Morfeld et al. 2015).

For the risk assessment of nanomaterials, it is relevant that the tumor formation observed for MG4 high surface carbon black and TiO_2_ in rats does not originate from primary genotoxic effects and that, hence, a threshold could be defined. Further investigations are necessary to determine the relevance of tumorigenic effects in rats for humans, just as the biokinetics and dosimetry of such effects in rats need to be better understood and characterized. Eventually, the outcome of the ongoing long-term inhalation study (Landsiedel et al. 2016; Ma-Hock et al. 2016; Groeters et al. 2017) will help provide a basis for the risk assessment of nanomaterials.

## Nanomaterial hazard assessment upon oral, dermal, or local exposure

The DF4nanoGrouping case studies addressed inhalation as the primary route of exposure for many nanomaterials. Thereby, they focused on potential human health effects in the respiratory tract as the primary target organ upon inhalation as well as in secondary organ systems that might be affected if nanomaterials become systemically available after deposition in the lung. Nevertheless, the general approach of the DF4nanoGrouping is equally applicable to other routes of exposure, such as *oral or dermal exposure or local exposure to the eyes*. Generally, nanomaterials are of low systemic availability upon oral exposure and do not elicit apical effects (Buesen et al. 2014). Also, the few available oral reproductive toxicity studies do not point to effects on fertility or development of, e.g., SiO_2_ NM-200 (Hofmann et al. 2015; Wolterbeek et al. 2015). Similarly, the available in vitro and in vivo studies do not report unintentional permeability or systemic availability of dermally applied nanomaterials, such as nanomaterials used in sunscreen lotions (Monteiro-Riviere et al. 2011; Landsiedel et al. 2012b). Finally, a broad spectrum of nanomaterials did not exhibit eye irritation potential in the EpiOcular™ eye irritation test (OECD TG 492) or the Bovine Corneal Opacity and Permeability (BCOP; OECD TG 437) (Kolle et al. 2016). Only a silver nanomaterial supplied as dispersion tested positive in the EpiOcular™ eye irritation test, and it produced highly variable results in the BCOP assay with dark-brown patches remaining on the corneal surface (Kolle et al. 2016). Chronic human exposure to silver has been reported to elicit permanent bluish-gray discoloration of the eyes (SCENIHR 2015).

## Application of the DF4nanoGrouping for read-across

One of the challenges in developing efficient risk assessment approaches for nanomaterials is to make the best use of the still scarce available data on physico-chemical properties, exposure, toxicokinetics, fate, and hazard (Oomen et al. 2015). Grouping and read-across approaches for nanomaterials can help to streamline this best use of information (Oomen et al. 2015). The DF4nanoGrouping provides a structured framework to substantiate the application of read-across approaches, and the comprehensive database available for the 25 case study substances (Arts et al. 2016) renders them suitable source substances for read-across. As described in the RAAF (ECHA 2017), all read-across approaches should begin with the formulation of a specific hypothesis, e.g., ‘the target substance has the same type of effect as the source substance,’ and any given read-across prediction can only be made for a specific toxicological endpoint.

All Tiers 1–3 essential grouping criteria of the DF4nanoGrouping data can be selected as specific toxicologically relevant properties to generate a hypothesis for read-across. The essential grouping criteria ‘water solubility’ and ‘dissolution in biological media’ can be selected as properties to identify MG1 soluble nanomaterials. The further risk assessment of MG1 soluble nanomaterials should be based on an evaluation of the effects of the released ions, which in turn may be undertaken by applying read-across approaches.

The essential grouping criteria ‘particle size and shape (aspect ratio)’ and ‘dissolution/biopersistence’ can be selected as properties to identify MG2 biopersistent HAR nanomaterials. The further risk assessment of these substances may be based on read-across to other biopersistent fibers.

For all nanomaterials that are neither MG1 nor MG2, the Tier 2 essential grouping criteria ‘surface reactivity,’ ‘dispersibility,’ and ‘cellular effects’ are useful properties to compare the respective DF4nanoGrouping MG3 and MG4 case study substances (i.e., source substances) to the target substance under investigation. For all nanomaterials that are predicted as MG4 ‘active,’ further testing in regard to toxic potency and biopersistence is necessary. The STIS is an appropriate test method to identify the specific NOAEC range as well as the likelihood of biopersistence and extra-pulmonary translocation (inside and outside the MPS) and the evolvement of pulmonary or systemic effects.

As required by the REACH Regulation (EP and Council of the EU 2006), NOAEC values obtained in toxicity studies are applied to calculate derived-no-effect-levels (DNELs) during risk assessment. Thereby, aerosol concentrations, above which humans should not be exposed, are determined. Specifically for workers, *Council Directive 98/24/EC on the protection of the health and safety of workers from the risks related to chemical agents at work* (Council of the EU 1998) requires the determination of occupational exposure limits (OELs) to prevent workers from being exposed to hazardous substance concentrations (Hristozov et al. 2016). With regard to dust, the German Federal Institute for Occupational Safety and Health sets a general threshold limit value (BAuA 2014). Assignment of a read-across target substance to either MG3 or MG4 may serve to determine whether the general threshold limit value for dust is applicable to ensure occupational safety upon long-term exposure to a given nanomaterial or not. The passive nanomaterials (MG3) are those for which the general threshold limit value for dust is sufficient, whereas active nanomaterials (MG4) are those that may require specific occupational exposure limits and, accordingly, specific further investigations, as relevant (Arts et al. 2015, 2016).

Since only few (representative) passive and active nanomaterials are being tested in long-term inhalation studies, read-across approaches are especially important for the prediction of long-term effects of target substances. To enable such predictions, knowledge on the kinetics of lung burden when exposure durations and observations periods exceed those applied in the STIS (5-day exposure; approx. 21 days post-exposure observation) is necessary. This is highlighted by the example of the MG3 passive nanomaterial BaSO_4_ NM-200. Upon inhalation exposure to 50 mg/m^3^, rat lung burdens were comparatively low (1 mg/g lung tissue) within the first 13 weeks of exposure and steeply increased to >10 mg per lung after 1 year, accompanied by severe inflammatory changes (Landsiedel et al. 2016; Groeters et al. 2017). Even though the very high aerosol concentrations of 50 mg/m^3^ do not call the assignment of BaSO_4_ NM-200 as MG3 passive into question, these observations underline the need to understand the kinetics of lung burden upon long-term exposure.

## Discussion

Compared to non-nanosized chemicals, nanomaterials appear to induce relatively few toxic mechanisms or potential AOPs. There is no evidence of ‘nanospecific’ mechanisms of action, and no step-change in hazard has been observed for particles below 100 nm (Moreno-Horn and Gebel 2014). Instead, the hazards of nanomaterials are dominated by fiber- or particle-like effects (reflected in the DF4nanoGrouping main groups MG2 biopersistent HAR nanomaterials and MG4 active nanomaterials, respectively) or by the effects of released ions (MG1 soluble nanomaterials).

Consisting of three tiers to assign nanomaterials to four main groups, with possible further subgrouping to refine specific information needs, the DF4nanoGrouping is an effective and efficient hazard and risk assessment tool that applies modern toxicology and contributes to the sustainable development of nanotechnological products. It allows rapid material categorization according to hazard potential, founded on scientifically justifiable categories, so that materials of high concern can be targeted for additional scrutiny, while material categories that pose the least risk can receive expedited review (Godwin et al. 2015; Dekkers et al. 2016).

In the case studies putting DF4nanoGrouping into practice (Arts et al. 2016), 22 of the 25 test materials fitted into the four MGs based on the non-animal Tiers 1 and 2 alone. For the other three materials, the hazard was overpredicted in the non-animal tiers, i.e., they indicated a concern that was not confirmed in Tier 3 in the in vivo STIS:

- SiO_2_.phosphate (an amorphous SiO_2_ with negatively charged phosphate surface functionalization; primary particle size 15 nm): In Tier 2, its high dispersibility (in Dulbecco’s Modified Eagle Medium with 10% fetal calf serum) indicated concern. In Tier 3, a high STIS NOAEC (>50 mg/m^3^) and a lack of extra-pulmonary translocation or systemic alterations (recorded either clinically or during histopathological evaluation) led to the conclusion that SiO_2_.phosphate is a MG3 passive nanomaterial.
- Organic Pigment blue 15:1: In Tier 2, its activity in the in vitro alveolar macrophage assay indicated concern. In Tier 3, its STIS NOAEC (≥30 mg/m^3^) led to the conclusion that Pigment blue 15:1 is a MG3 passive nanomaterial.
- Graphite nanoplatelets: In Tier 2, data on surface reactivity were unavailable for technical reasons. Hence, surface reactivity could not be excluded. In Tier 3, the STIS NOAEC (≥10 mg/m^3^) led to the conclusion that graphite nanoplatelets are MG3 passive.

Possibly, the dispersibility of nanomaterials in the culture medium is altered by surface functionalization, e.g., the dispersibility of SiO_2_ without surface functionalization lies above the threshold (average agglomeration number (AAN) <3; Tables 1 and 2), whereas the dispersibility of SiO_2_.acrylate and SiO_2_.phosphate lies below this threshold. Indeed, for SiO_2_.acrylate, extra-pulmonary translocation to the spleen (i.e., inside the MPS) was recorded. However, as the example of SiO_2_.phosphate shows, dispersibility with low AAN does not necessarily result in extra-pulmonary translocation and the resulting potential to elicit systemic alterations. The example of Pigment blue 15:1 may point to a higher sensitivity of the in vitro alveolar macrophage assay as compared to the in vivo STIS. However, for precautionary reasons such a higher sensitivity is to be welcomed. The example of graphite nanoplatelets shows that if a specific grouping criterion cannot be determined for technical reasons, concern (MG4) has to be assumed for precautionary reasons.

Avramescu et al. (2016) applied the DF4nanoGrouping to assess the influence of pH value, particle size, and crystal form on the dissolution behavior of zinc metal, ZnO, and TiO_2_ nanomaterials and their bulk counterparts in biological media. Avramescu et al. (2016) concluded that the DF4nanoGrouping is applicable but reported that data cannot (always) be taken from tabulated sources but need to be determined for the specific materials at known and relevant pH to enable correct comparison and grouping.

An asset of DF4nanoGrouping is that it places emphasis on system-dependent effects (the three Tier 2 grouping criteria: dissolution in biological media, surface reactivity, and dispersibility). Thereby, DF4nanoGrouping can be applied to test materials in different media or to the as-released forms, e.g., particulate fragments from CNT-containing composites or fragments released from occupational manufacturing and handling of non-nano materials (Wohlleben et al. 2011a; Saber et al. 2016). Furthermore, DF4nanoGrouping can be applied to non-nanosized materials, as the comparative assessment of nanosized and non-nanosized diketopyrrolopyrrol orange showed (Arts et al. 2016).

The DF4nanoGrouping was developed oriented towards EU legislation and specifically the REACH Regulation. Notwithstanding, its grouping criteria and its four main groups are sufficiently general to allow using the DF4nanoGrouping in other jurisdictions as well. In July 2016, the DF4nanoGrouping has been specifically recommended by Environment and Climate Change Canada and Health Canada in their prioritization approach for nanomaterials: The scheme proposed for ranking human health hazard according to nanomaterial properties is modeled after the DF4nanogrouping approach, which groups nanomaterials according to their specific mode of action that may result in a toxicological effect. The approach considers both physical-chemical properties and system-dependent properties. This approach is also aligned with the approaches put forth by the RCC Nano [Canada-US Regulatory Cooperation Council nanotechnology work plan], which establishes key criteria for identifying key characteristics of nanomaterials, and presents a framework for assessing and identifying testing requirements for nanoparticles (ECCC and HC 2016).

## Conclusion

In case studies covering 25 test materials, the DF4nanoGrouping proved to be an effective and efficient hazard and risk assessment tool that applies modern toxicology and contributes to the sustainable development of nanotechnological products.

The DF4nanoGrouping may be applied and further developed at the same time making use of new knowledge on the relationship between intrinsic and system-dependent properties as it becomes available. A circumscribed number of grouping criteria has been identified as essential for the DF4nanoGrouping, and these criteria are linked by ‘and’ and ‘or’ relationships. As further knowledge on how nanomaterials interact with their environment becomes available, the suitability of these grouping criteria may be confirmed or refuted, the current threshold values may be adapted, and additional grouping criteria may be identified. Further, it may become possible to identify more complex relationships and interdependencies between different nanomaterial properties that may be used to refine the DF4nanoGrouping. The identification of relevant grouping criteria also depends on the availability of relevant and reliable methodologies to assess the respective properties. Future research should be directed at both goals, i.e., at enhancing knowledge on the interaction of nanomaterials with different environments, also by submitting the DF4nanoGrouping to further case studies, and at making improved methodologies available for the assessment of relevant nanomaterial properties.

Nanomaterial assignment to one of the four MGs of the DF4nanoGrouping further provides preliminary information on the mode-of-action of nanomaterials. Building knowledge about the modes-of-action of toxicological effects of different nanomaterials (different nanomaterials will employ different modes-of-action and a given nanomaterial may attend in more than one mode-of-action) will enable informed, evidence-based in vitro models to be identified, which can be used in the first instance to screen for apical toxic effects and which may reduce the number of nanomaterials taken forward for in vivo testing (Burden et al. 2017).

Future research should aim at making a decision tree available on how the DF4nanoGrouping may be integrated into the REACH registration process for substances that have to be registered in the nanoform. Finally, the DF4nanoGrouping case studies (Arts et al. 2016) may also form the scientific basis for the justification of read-across applications, e.g., by using the DF4nanoGrouping benchmark materials as source nanomaterials for read-across (Teubner and Landsiedel 2015). Altogether, the advanced ‘multiple perspective’ decision-making framework DF4nanoGrouping, that is closely linked to IATAs, ensures that no studies are performed that do not provide crucial data and that therefore would lead to a waste of animals and resources (Oomen et al. 2014a; Arts et al. 2014, 2015, 2016).

## Footnotes

1 50 mg/m^3^; inhalation for 1800 min (6 h/day on 5 days) at 0.2 L/min (Snipes 1988).
